# The method for measurement of the three-dimensional scoliosis angle from standard radiographs

**DOI:** 10.1186/s12891-020-03494-w

**Published:** 2020-07-21

**Authors:** Paweł Główka, Wojciech Politarczyk, Piotr Janusz, Łukasz Woźniak, Tomasz Kotwicki

**Affiliations:** 1 Department of Spine Disorders and Pediatric Orthopedics, Poznan University of Medical Sciences, 28 Czerwca 1956 135/147, 61-545 Poznan, Poland; 2 Institute of Mathematics, Warsaw University, Banacha 2, 02-097 Warsaw, Poland; 3 Department of Pediatric Orthopedics and Traumatology, Poznan University of Medical Sciences, 28 Czerwca 1956 135/147, 61-545 Poznan, Poland

**Keywords:** Scoliosis, 3D scoliosis angle, Three-dimensional evaluation of scoliosis, Three-dimensional idiopathic scoliosis angle

## Abstract

**Background:**

Three-dimensional idiopathic scoliosis cannot be accurately assessed with the aid of a single plane parameter – the Cobb angle. We propose a novel method for evaluating the three-dimensional (3D) pattern of scoliosis based on two X-rays (PA and lateral). The proposed method consists of the measurements of the angles between the upper endplate of the upper-end vertebra and the lower endplate of the lower-end vertebra (3D scoliosis angle).

**Methods:**

The 3D-angles of thirty scoliosis curves were measured with either computed tomography (CT) or digitally reconstructed radiographs (DRRs): PA and lateral. CT was used as a reference. In the case of CT, the 3D angle was calculated based on the coordinates of three points situated on the upper endplate and those of three points situated on the lower endplate of the scoliosis curve. In the case of the DRR, the 3D angle was calculated using the four-angle method: the angles formed by the endplates of the curve with the transverse plane. The results were tested with the Student’s t-test, and the agreement of measurements was tested with the intraclass correlation coefficient.

**Results:**

There was no significant difference between the 3D-angle measurements obtained with DRRs versus CT, *p* > 0.05. There was, however, a significant difference between the 3D-scoliosis angle and the Cobb angle measurements performed based on the X-rays. The reproducibility and reliability of 3D angle measurements were high.

**Conclusions:**

Based on two standard radiographs, PA and lateral, it is possible to calculate the 3D scoliosis angle. The proposed method facilitates 3D-scoliosis assessment without the use of sophisticated devices. Considering the 3D nature of AIS, the 3D parameters of the spine may help to apply a more effective treatment and estimate a more precise prognosis for patient with scoliosis.

## Background

Scoliosis is a three-dimensional deformity of the spine. The magnitude of scoliosis is typically measured with the Cobb angle on posteroanterior (PA) X-rays of the spine. The Cobb angle measured on the PA X-rays does not demonstrate the angle between the end vertebrae observed in three-dimensional (3D) space. The 3D character of scoliosis [1] renders the three-dimensional diagnostic evaluation preferable [2].

Studies have indicated that 3D scoliosis patterns can be predictive of deformity progression [3]. Two cases of scoliosis with similar two-dimensional morphologies may have different three-dimensional morphologies [4]. These findings emphasized the importance of 3D parameters. Despite a few parameters, the evaluation of scoliosis refers mainly to two two-dimensional planes: coronal and sagittal [5]. The evaluation of three-dimensional deformities has the following feasible parameters: the axial rotation of the vertebra, the orientation of the plane of maximum curvature (PMC) [4, 6], the angle of scoliosis observed in the PMC, and the top view parameters [7–9].

Nevertheless, two-dimensional X-ray evaluation [10, 11] has prevailed in the evaluation and follow-up of patients with adolescent idiopathic scoliosis (AIS). Presumably, this is because systems for the 3D analysis of scoliosis, such as the EOSTM imaging system [12, 13], are not widely accessible.

The increasing interest in the 3D parameters of scoliosis [3] and the wide use of X-rays for scoliosis evaluation have caught our attention in terms of the evaluation of the 3D character and magnitude of scoliosis. The primary objective of the study was to propose a novel method for calculating the 3D angle that exists between the upper endplate of the upper-end vertebra and the lower endplate of the lower-end vertebra (hereafter called the 3D scoliosis angle). The 3D scoliosis angle was evaluated based on two standard X-rays: PA and lateral. The secondary objective was to evaluate the accuracy, reproducibility and reliability of this method.

## Methods

The introduction and validation process of the method for 3D scoliosis angle calculations was accomplished in four steps: 1) calculation of the 3D scoliosis angle based on computed tomography (CT); 2) calculation of the 3D scoliosis angle based on digitally reconstructed radiographs (DRRs); 3) comparison of the 3D scoliosis angle calculations: CT versus DRRs; and 4) evaluation of the reproducibility and reliability of the proposed method based on X-rays (PA and lateral).

### Subjects

The study involved 41 patients with AIS. That population consists of two groups of patients. The first group of patients was involved in the first part of the study—the introduction and validation of the new method for the 3D evaluation of scoliosis.

The first group consisted of 10 patients with AIS scheduled for the surgery. Inclusion criteria: AIS; presence of a main curve: thoracic or lumbar; imaging modalities performed during the hospitalization: good quality plain-standing X-rays (PA and lateral); and CT of the thoracic and lumbar spine performed as a part of the presurgery protocol. Exclusion criteria: scoliosis other than the idiopathic type, a lack of CT or PA and lateral standing X-ray data, and poor-quality X-rays. Each patient had three scoliosis curves in the thoracolumbar region, yielding CT data of 30 scoliosis curves. The characterization of the first group of patients was as follows: mean age of 14 yo (range: from 10 to 17), mean body weight of 45.2 kg (range: from 28.0 to 65.0), mean BMI of 17.9 (range: from 14.8 to 22.5), mean scoliosis curve 52° (range: from 11° to 130°), and mean main curve 75° (range: from 51° to 130°).

The second group of the patients consisted of 31 patients with AIS. The second group was involved in the evaluation of the reproducibility and reliability of the proposed new measurement. The inclusion and exclusion criteria were the same as those for the aforementioned first group of patients with the exclusion of CT data of the spine. Each patient had at least two scoliosis curves in the thoracolumbar region: a main curve and a secondary curve, yielding 62 scoliosis curves. The characterization of the second group of patients was as follows: mean age of 15 yo (range: from 10 to 17), mean body weight of 54.9 kg (range: from 26.5 to 97.6), mean BMI of 20.0 (range: from 14.4 to 32.1), mean thoracic scoliosis curve of 65.6° (range: from 42.8° to 100.7°), mean lumbar or thoracolumbar curve of 44.2° (range: from 22.7° to 80.4°), and mean scoliosis curve (thoracic, thoracolumbar or lumbar) of 54.9° (range: from 22.7° to 100.7°). The magnitude of the scoliosis was measured with the Cobb method.

The CT scans of thirty scoliosis curves from patients with AIS were analyzed. The CT scans were not performed for the purpose of the study but as a part of the presurgery protocol. The CT scans were analyzed retrospectively with acceptance of the local Institutional Review Board. The CT scans were obtained in a supine position with the Siemens Emotion 16-row multidetector computer tomography. Data were stored in DICOM (Digital Imaging and Communications in Medicine) format files.

Standing X-rays (PA and lateral) of the full spine were obtained from a distance of 2 m. The radiograms were recorded in digital version in DICOM files.

### Calculation of the 3D scoliosis angle based on CT scans

**Fig. 1:**
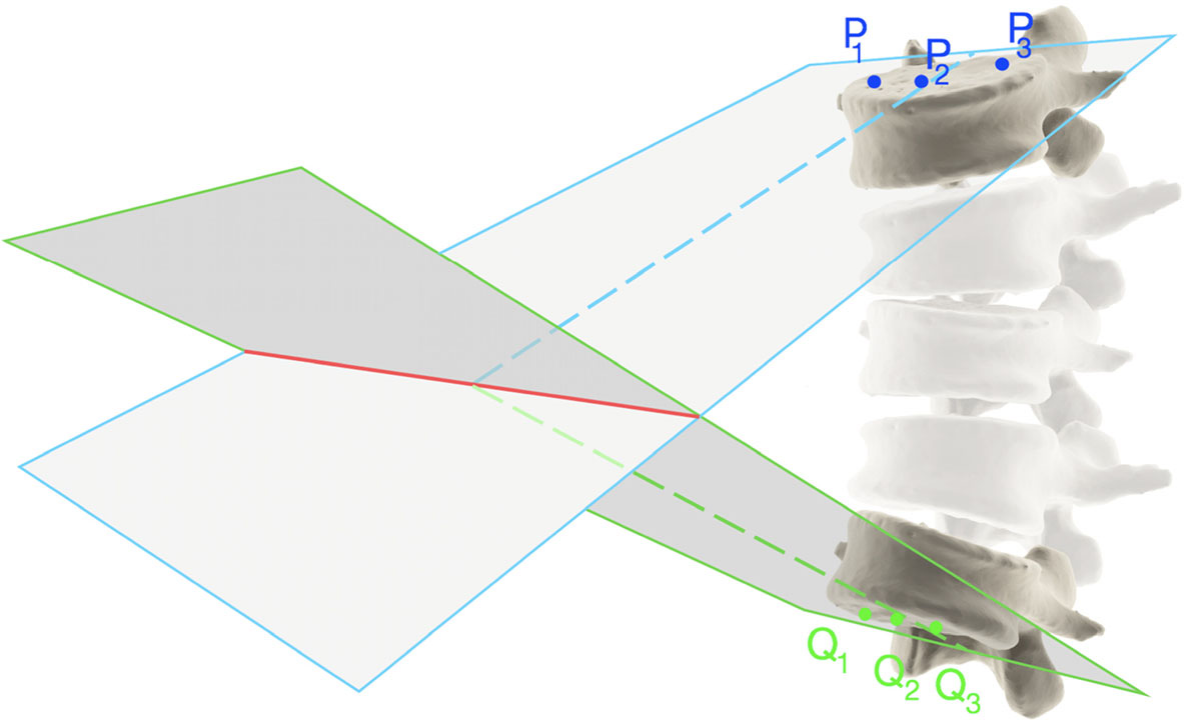
Triple-point method for evaluation of the angle between the upper and lower endplates of the scoliosis curve based on computed tomography scans. The blue plane is parallel to the upper-end plate of the upper-end vertebra. The green plane is parallel to the lower endplate of the lower-end vertebra. The angle between the intersecting (spotted) lines is an angle between the mentioned planes (3D-scoliosis angle)

As the first step, the CT scans of the patients were analyzed. The 3D scoliosis angle was calculated based on the coordinates of three points situated on the plane (π1) parallel to the upper endplate of the upper-end vertebra and on the coordinates of three points situated on the plane (π2) parallel to the lower endplate of the lower-end vertebra of the scoliosis curve (Fig. 1). The CT scans of the spine were analyzed with the DeVide software (The Delft University of Technology, The Netherlands). The software visualized the spine in three planes that intersected with each other. The angles between those planes could be manually adjusted. The axial plane was set up in such a manner that it was parallel to the upper endplate of the upper-end vertebra. The coordinates of three discretionary points lying in this plane were saved. Next, the axial plane was set up in such a manner that it was parallel to the lower endplate of the lower-end vertebrae. The coordinates of three discretionary points lying in this plane were saved. In this way, the three points lying on each endplates were defined. These points were used to calculate the angle between the planes in which they were situated.

### Calculation of the scoliosis angle based on digitally reconstructed radiographs (DRRs)

**Fig. 2:**
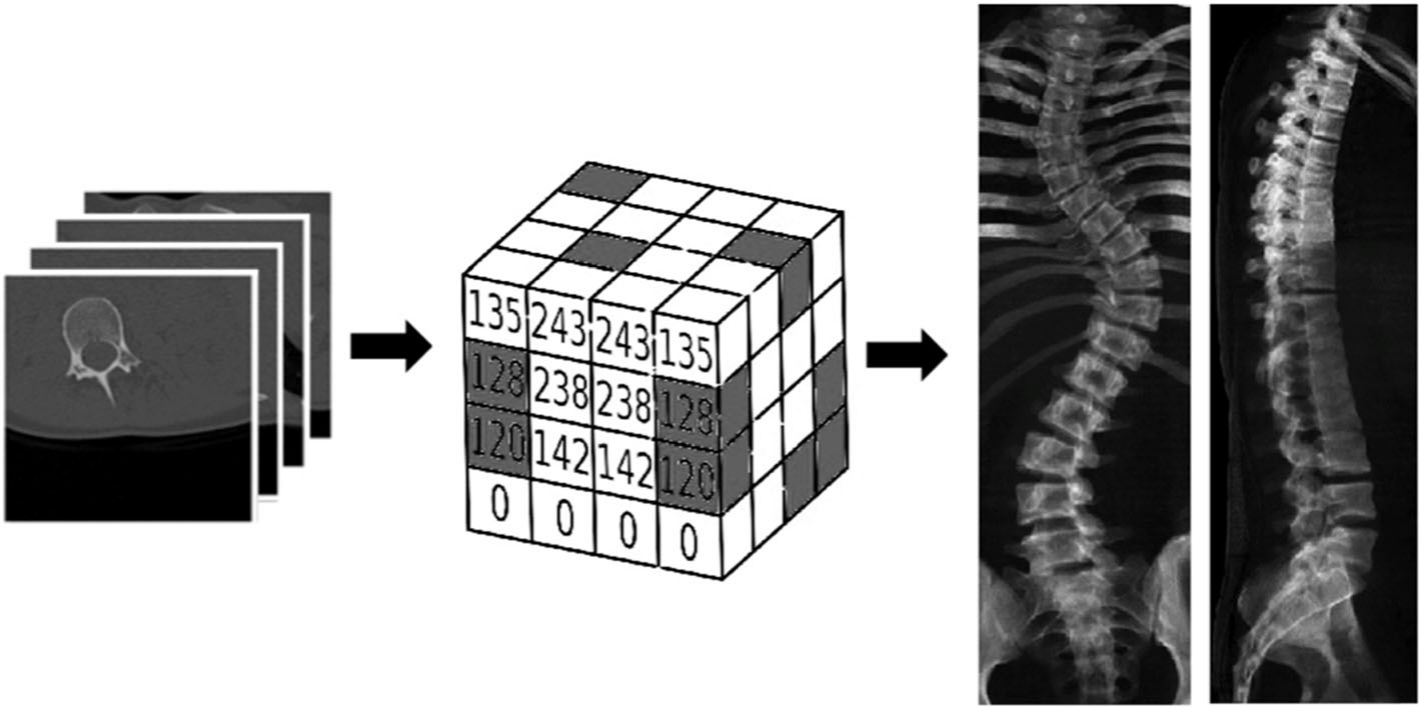
Schematic presentation of the production of digitally reconstructed radiographs from computed tomography scans

The DRRs were designed from the CT scans using the technique published by our team [14, 15]. First, CT DICOM images were converted into PNG file format. A 3D array of the grayscale values received from the CT images was created. Afterwards, a mean value of each x, y and z direction was calculated. The results were stored in 2D arrays representing three planes: coronal, lateral and axial. The 2D arrays were used for further calculations. Significance boundaries for each row and column were calculated with the aim of creating final DRRs. Afterwards, the global coordinate system was determined, and the results were converted into DICOM file format, allowing further measurements [15]. A schematic presentation of the production of DRRs from CT scans is presented in Fig. 2.

**Fig. 3:**
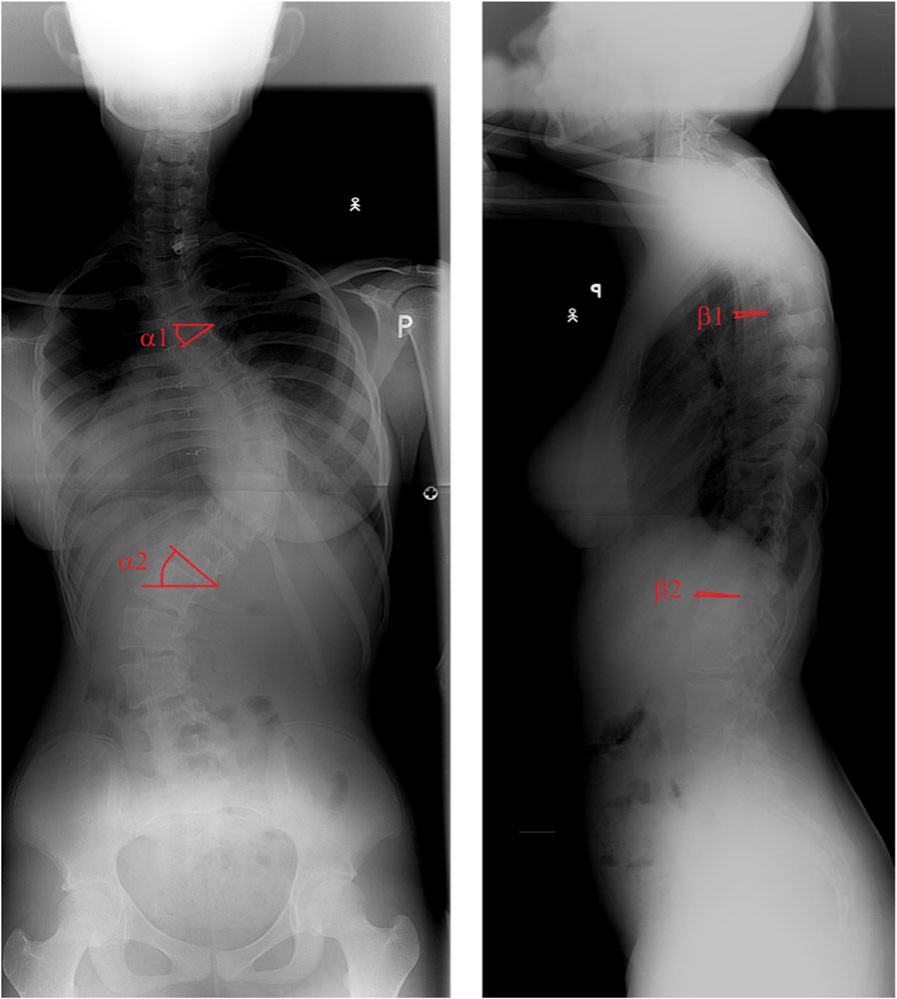
Four angle method for evaluating the angle between the upper and lower endplates of the scoliosis curve based on two X-rays scans: posterior-anterior and lateral

The angle between the endplates was measured as a dihedral angle. The dihedral angle is the angle between two intersecting planes [16]. The upper and lower endplates were approximated by two planes in a three-dimensional space. To measure angles between the planes, unit length normal (perpendicular) vectors of the respective planes were determined. The angle between the normal vectors within the plane spanned by these vectors was measured. Four angles were measured on the PA and lateral DRRs (four-angles method for 3D scoliosis angle calculation) (Fig. 3):

α_1_- the angle between the line parallel to the upper endplate of the upper-end vertebra and the transverse line measured in the coronal plane

α_2_- the angle between the line parallel to the lower endplate of the lower-end vertebra and the transverse line measured in the coronal plane

β_1_- the angle between the line parallel to the upper endplate of the upper-end vertebra and the transverse line measured in the sagittal plane

β_2_- the angle between the line parallel to the lower endplate of the lower-end vertebra and the transverse line in the sagittal plane.

These angles were used to calculate the angle between the endplates (_1_ and _2_) using the following mathematical formula:

$$ \frac{180}{\pi }.\kern0.5em \operatorname{arccos}\kern0.5em \left(\frac{T_1\kern0.5em .\kern0.5em {U}_{1\kern0.5em }+\kern0.5em {T}_2\kern0.5em .\kern0.5em {U}_2\kern0.5em +\kern0.5em {T}_3\kern0.5em .\kern0.5em {U}_3}{\sqrt{T_1^2\kern0.5em +\kern0.5em {T}_2^2\kern0.5em +\kern0.5em {T}_3^2\kern0.5em .\kern0.5em \sqrt{U_1^2\kern0.5em +\kern0.5em {U}_2^2\kern0.5em +\kern0.5em {U}_3^2}}}\right) $$

Define

$$ {T}_1\kern0.5em =\kern0.5em \sin \kern0.5em \left({a}_1\right)\kern0.5em .\kern0.5em \cos \kern0.5em \left({\beta}_1\right) $$

$$ {T}_2\kern0.5em =\kern0.5em \sin \kern0.5em \left({a}_1\right)\kern0.5em .\kern0.5em \cos \kern0.5em \left({\beta}_1\right) $$

$$ {T}_3\kern0.5em =\kern0.5em \sin \kern0.5em \left({a}_1\right)\kern0.5em .\kern0.5em \cos \kern0.5em \left({\beta}_1\right) $$

$$ {U}_1\kern0.5em =\kern0.5em \sin \kern0.5em \left({a}_2\right)\kern0.5em .\kern0.5em \cos \kern0.5em \left({\beta}_1\right) $$

$$ {U}_2\kern0.5em =\kern0.5em \sin \kern0.5em \left({a}_2\right)\kern0.5em .\kern0.5em \cos \kern0.5em \left({\beta}_1\right) $$

$$ {U}_3\kern0.5em =\kern0.5em \sin \kern0.5em \left({a}_2\right)\kern0.5em .\kern0.5em \cos \kern0.5em \left({\beta}_1\right) $$

### Comparison of the results of 3D scoliosis angle calculations: CT versus DRRs

The results of the measurements of the 3D scoliosis angle based on the CT scans and DRRs were tested with paired Student’s t-tests. A *p* level of 0.05 was considered significant. The power of the t-test was set at 0.95.

### Comparison of the results of 3D scoliosis angle calculations and Cobb angle measurements based on X-rays

The 3D scoliosis angle was calculated based on two X-rays, PA and lateral, with the *four-angle method* described above. The Cobb angle was measured on the PA X-ray. The results of the 3D-scoliosis angle calculations and Cobb angle measurements were tested with the paired Student’s t-test.

The reliability and reproducibility of the 3D scoliosis angle measurements were tested with the use of PA and lateral X-rays of 31 patients, which yielded 62 curves in total. Data from anonymous X-rays were used and were evaluated by two independent observers: a spine surgeon, and a resident in orthopedics in the fifth year of residency. The first observer performed the measurements once, and the second observer performed the measurements twice with a two-week interval between measurements. The reproducibility and reliability of the measurements were tested with the intraclass correlation coefficient (ICC).

The CT scans, DRRs and X-rays were anonymized and presented to the readers in random order.

### Statistical analysis

The data were analyzed using the Statistica (StatSoft) and Microsoft Office Excel (2018 Microsoft). Normal distribution of data was tested by use of Shapiro-Wilk test. Paired Student’s t-tests were used to test the differences for the continuous data. A *p* level of 0.05 was considered significant. The power of t test was set at 0.95. Intraobserver reproducibility and intraobserver reliability were tested with the ICC. To estimate the sample size required to test the intraobserver reproducibility and intraobserver reliability of the measurements, we treated an ICC value greater than 0.7 (with its 95% confidence interval of 0.55–0.85) as acceptable reproducibility for the research tool [17, 18]. The minimum number of subjects to test the agreement, intraobserver reproducibility and interobserver reliability was 44 [19]. The number of 62 scoliosis curves was sufficient for ICC calculation.

## Results

**Fig. 4:**
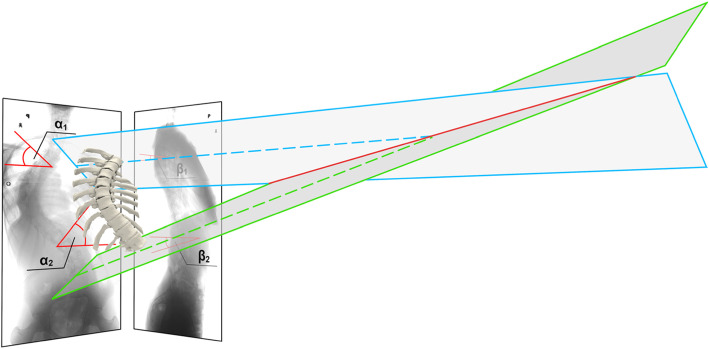
The angle between the intersecting (spotted) lines is the 3D-scoliosis angle

The results of the 3D scoliosis angle (Fig. 4) calculations made based on the CT scans and DRRs (PA and lateral) are shown in Table 1. There was no significant difference between the measurements of the 3D scoliosis angle calculated based on measurements obtained with CT versus DRR (Table 1).

**Table 1:** 3D scoliosis angle calculated based on CT versus DRR, *n* = 30

	CT			DRR			Difference		t test
	mean	SD	range	mean	SD	range	mean	SD	*p*
angle [°]	31.21	23.02	7.09–131.13	31.68	22.80	9.22–130.99	0.88	0.87	0.19

*CT* computed tomography, *DRR* digitally reconstructed radiograph, *SD* standard deviation

There was a significant difference between the 3D scoliosis angle and the Cobb angle measurements performed based on X-rays. The results are illustrated in Table 2.

**Table 2:** Comparison of the Cobb angle and 3D scoliosis angle calculate based on X-rays, *n* = 62

	3D-scoliosis angle			Cobb angle			Mean difference		T-test
	mean	SD	range	mean	SD	range	mean	SD	*P*
angle [°]	60	15	35–105	54	17	22–101	5	4	< 0.0001

The ICCs for the interobserver reliability and the intraobserver reproducibility with *p* = 0.05 for the 3D scoliosis angle calculated with two X-rays (PA and lateral) were high. The results are presented in Table 3.

**Table 3:** ICC for the 3D scoliosis angle and Cobb angle calculated based on X-rays, *n* = 62

	ICC	
	Intra	Inter
3D-scoliosis angle	0.99	0.93
Cobb angle	0.98	0.91

## Discussion

3D parameters of the spine have been increasingly used in AIS for the evaluation of the severity of scoliosis, surgical outcomes, and curve progression [3]. Some authors have attempted 3D scoliosis angle measurements. Stagnara introduced a particular form of spine projection presenting scoliosis in the plane of maximum curvature [20]. To obtain this projection, the photographic X-ray plate is placed in the plane, which is rotated in relation to the coronal plane around the longitudinal axis of the spine.

The development of new diagnostic tools, such as CT, MRI, and EOS, made the 3D analysis of the spine morphology feasible. CT remains the gold standard for bone morphology visualization. Nevertheless, it exposes patients to a high dose of radiation, which makes CT unsuitable for routine use in patients with scoliosis. MRI remains a preferred method for soft tissue visualization. Both CT and MRI are performed in the lying position, which diminishes the influence of gravity on the spine. Scoliosis is a load-bearing deformity, so the follow-up of patients with scoliosis involves standing X-rays. The EOS imaging system enables the visualization of the spine in the standing position with a low-dose X-ray scanning technique [12, 13], but this method is not widely accessible.

This study presents a novel method that facilitates the evaluation of the angle between the endplates of the upper and lower end vertebrae based on PA and lateral X-rays called the 3D scoliosis angle (Fig. 4). The angle presents an inclination angle between the vertebrae.

The proposed method does not require any sophisticated devices or software, but two X-rays of the spine (PA and lateral) in the standing position. The 3D scoliosis angle can be calculated based on four angles measured on PA and lateral X-rays, as presented in the Methods (Figs. 3, 4).

The method for 3D-scoliosis angle measurement was validated with CT scans as the gold standard. The X-rays are taken in the standing position, and CT is performed in the lying position. X-rays demonstrate scoliosis with the influence of gravity. For this reason, measurements and calculations performed based on X-rays (standing position) cannot be compared with measurements performed based on CT (lying position). This different position encouraged us to develop something that we could use instead of X-rays in the validation process. We created DRRs from CT scans [14, 15]. DRRs and CT scans demonstrate the spine in the same position. DRRs replaced X-rays in the validation process.

The results showed no significant difference in measurements of the 3D scoliosis angle performed based on CT scans and DRRs, which indicates that the introduced four-angle method can be used for 3D scoliosis angle evaluation with X-rays.

The assessment of the 3D real angle based on a two X-rays is not a new idea. Dunn, Rippstein, and Muller introduced the method for the radiological assessment of the real femoral neck-shaft angle and real femoral anteversion angle based on two X-rays of the hip. The method involves measurements of the projected anteversion angle and projected neck-shaft angle on conventional X-rays. Today, the Dunn-Rippstein-Müller method [21] is widely used to assess the rotational deformities of the proximal femur.

A limitation of the study is that the study group consisted of patients with severe scoliosis. CT exposes patients to a large dose of radiation, so due to ethical reasons, CT scans were not ordered for patients with small curvatures. Another limitation of the study is the fact that CT measurements are not comparable in a simple way with the measurements performed on X-rays. CT and X-rays demonstrate the spine in different positions; therefore, we could not compare the results of measurements performed based on CT vs X-rays, in the validation process. Instead, we decided to create DRRs that demonstrated the spine in the same position as CT.

There are some confounding factors that might preclude the use of the proposed method: patient obesity, previous surgery with the spine implants, and poor-quality X-rays. All of these factors could influence the sharpness of the vertebrae and blur the spine on X-rays. Spine implants can additionally obliterate the endplates of the vertebrae, rendering measurements impossible. Scoliosis follow-up requires PA X-rays of the spine in half-year intervals. The proposed method requires two X-rays, PA and lateral, which increases the patient’s radiation dose. All of these limitations may limit the use of the method to selected cases.

We believe that the introduced 3D scoliosis angle measurement more closely reflects the real relationships among vertebrae in scoliosis curves than the scoliosis angle measured on X-rays in the coronal plane alone.

Considering the 3D nature of AIS, the 3D parameters of the spine may help to apply a more effective treatment and estimate a more precise prognosis for each patient with spine deformation.

## Conclusions

Based on two standard radiographs, PA and lateral, it is possible to measure the angle that develops in the space between the upper endplate of an upper-end vertebra and the lower endplate of a lower-end vertebra (3D scoliosis angle). The 3D scoliosis angle may provide clinicians with additional information on the morphology of the scoliosis deformities.

## Data Availability

The datasets analysed during the current study are available from the corresponding author on reasonable request.

## Abbreviations

PA: Posteroanterior
3D: Three dimensional
CT: Computed tomography
DRRs: Digitally reconstructed radiographs
AIS: Adolescent idiopathic scoliosis
DICOM: Digital Imaging and Communications in Medicine
MRI: Magnetic resonance imaging
